# The Lucerne Groove Library: An audio stimulus corpus of 444 short Western popular music drum and bass patterns with behavioural, structural, and audio measurements

**DOI:** 10.3758/s13428-026-02989-z

**Published:** 2026-05-05

**Authors:** Toni A. Bechtold, Rafael Jerjen, Florian Hoesl, Lorenz Kilchenmann, Olivier Senn

**Affiliations:** 1 Lucerne School of Music, Lucerne University of Applied Sciences and Arts, Arsenalstrasse 28a, 6010 Kriens, Switzerland; 2 Department of Music, University of Birmingham, Birmingham, UK

**Keywords:** Groove, Music psychology, Stimulus set, Drums, Bass

## Abstract

This study presents an audio stimulus corpus consisting of 444 Western popular music drum and bass patterns called the Lucerne Groove Library. Common requirements for stimuli in music psychological research, particularly studies on the experience of groove, are outlined, followed by a description of how these criteria are addressed in the presented corpus. For example, the corpus is designed to combine ecological validity with high manipulability, facilitating the use of the corpus in a variety of experimental settings. The methods section provides a detailed account of material selection, audio creation, and measures. Ground-truth behavioural data for the stimuli were obtained through a listening experiment (e.g., participant's ratings on the urge to move in response to the stimuli, and style assignments ). Several structural data (e.g., tempo and event density) and audio features (e.g., low-frequency sub-band flux) were measured for each stimulus, and an overview over all data is provided. Potential applications of the corpus in future studies are discussed, including variables along which stimuli can be selected or manipulated. The corpus is published in different formats and with a range of accompanying data, all of which can be found in the online repository.

## Introduction

“Groove is when music makes me move.” This simple definition of groove by a musician in a recent interview study (Bechtold et al., 2023, p. 358) situates the groove experience at the core of a fundamental human response to music: hundreds of millions of people move their bodies to music each day, whether at home, on a bus, in a club, or at a concert; alone or with others. Movement types range from head bobbing or finger tapping to full-body dancing. Given the ubiquity of music-related body movement in contemporary society, it is not surprising that the study of groove (i.e., the investigation of why the movement occurs) has garnered considerable attention from researchers in the last two decades. A fast-growing body of work (see Etani et al., 2024, for an overview) examines groove from different angles and with different methodologies drawn from psychology, neuroscience, physiology, or sociology. What most studies have in common is that they investigate the groove experience by letting listeners respond to musical audio stimuli. The optimal choice of musical stimuli is not trivial: researchers aim to control the dimensions along which the stimuli vary while preserving the ecological validity of the music. Researchers may want to use music from diverse musical genres or stimuli that represent diversity within a genre. They may need custom-tailored stimuli sets for specific purposes or sets that are used more widely and allow for better comparability across studies. This study presents a new stimulus set that aims to satisfy many of these needs and may be applied in a wide range of groove research settings.

Groove has been defined as a pleasurable urge to move along to music (Janata et al., 2012), often operationalized as a two-dimensional psychological concept involving pleasure and urge to move (e.g., Witek et al., 2014; Senn et al., 2020; Düvel et al., 2021; Kawase et al., 2025). Recent qualitative studies found that the groove experience has more dimensions to it, such as participation, immersion, and social aspects (Bechtold et al., 2023; Duman et al., 2024), and that the actual groove experience varies between individuals (Hosken, 2020) and has many facets.

Most studies about groove, however, examined characteristics of music that motivate listeners to move along. While early studies or analyses used commercial full-band music (Danielsen, 2006; Iyer, 2002; Madison, 2006; Madison et al., 2011; Witek, 2009), further investigations quickly turned to de-contextualized musical patterns with a reduced number of instruments. This can be exemplified with two “hot topics” within groove research: microtiming and syncopation. The investigation of microtiming as a factor affecting the groove experience was carried out on the basis of stimuli that allow for timing manipulations (Butterfield, 2010; Datseris et al., 2019; Davies et al., 2013; Frühauf et al., 2013; Kilchenmann & Senn, 2015; Senn et al., 2016). The investigation of syncopation or complexity as a predictor for groove was predominantly investigated on the basis of drum beats (Senn et al., 2018, 2024; Spiech et al., 2022; Witek et al., 2014), although some used other instruments for that purpose as well (Madison & Sioros, 2014; Sioros et al., 2014). Both strands share a common focus on rhythm over other musical parameters. This tendency can be justified with accounts that link rhythm perception with motor entrainment (Large & Jones, 1999; Large & Snyder, 2009), neural entrainment to a beat (Nozaradan et al., 2011), or the general role of the motor system for rhythm perception (Grahn, 2012). Based on such fundamental frameworks, many theories and investigations in groove research rely on rhythm or meter (Madison et al., 2011; Stupacher et al., 2022; Witek et al., 2014, 2017), which may explain why drum patterns are very frequently used as stimuli (Kawase & Eguchi, 2010; Etani et al., 2018; Eaves et al., 2019; Bechtold et al., 2024, 2025a; Seeberg et al., 2024). Only a few studies emphasized other parameters, e.g., harmonic complexity (Matthews et al., 2019), timbral features (Bechtold et al., 2025b, 2025c; Burger et al., 2013; Stupacher et al., 2016), or tempo (Etani et al., 2018; Jerjen et al., preprint).

Many stimuli that are used in groove studies and that systematically vary one or more specific musical aspects have a synthetic sound. They often contain simple and repeated MIDI samples and omit common steps of music production, such as sound design, mixing, or adding reverb—even though such production aspects are of utmost importance to music creators (Bechtold et al., 2023). Often, the stimuli show no variation in sound, no room simulation, and a lack of dynamics (e.g., Davies et al., 2013; Matthews et al., 2019; Spiech et al., 2022; Witek et al., 2014). In consequence, the stimuli may sound dull or artificial to listeners. In many studies, experimental control takes precedence over ecological validity with unknown consequences for the validity of the study results.

The use of the word ‘groove’ with respect to music has its roots in Afro-American culture (Câmara & Danielsen, 2018). Accordingly, most groove studies are conducted in the context of Western popular music. This is not surprising, considering that music consumption is dominated by popular music styles in Western societies, especially during dance- or movement-related activities (clubbing, sports). The mentions of other genres in groove research are sporadic, e.g., a listing of similar concepts in other genres (Madison, 2006; Senn et al., 2018) or a single comment about groove in classical music in Bechtold et al. (2023). Western popular music is a vast area; it encompasses countless genres and styles, but some have been shown to be associated closely with groove: funk, pop, and electronic dance music (EDM) (Senn et al., 2021; Stupacher et al., 2023; Witek et al., 2025). Popular music creators of a variety of musical styles have the explicit intention of making listeners move (Bechtold et al., 2023). However, many groove studies use small sets in which genres are only represented by a few songs, if at all. Hence, the variety within genres is not accounted for, and any relationship between a genre and groove might depend on the idiosyncrasy of the selected songs. This problem is further complicated by the fact that taste is important for experiencing an urge to move (see below).

In the past decade, several studies found that a listener’s musical background is a decisive factor for their groove response. Listeners’ experience is moderated by their familiarity with the music and music preferences (Bechtold et al., 2024, 2025a, b; Senn et al., 2018, 2021, 2023a). Dance preferences (Bechtold et al., 2024; Senn et al., 2024; Witek et al., 2014) and musical expertise or training—perhaps partially due to individual differences in beat perception or beat-finding abilities (Grahn & Schuit, 2012; Honing, 2012)—may also play a role, but results are not conclusive. Some studies found an effect of musical training or expertise on groove (Bechtold et al., 2024; O’Connell et al., 2022; Senn et al., 2018; Spiech et al., 2022; Stupacher et al., 2013), while others did not (Cameron et al., 2022a; Frühauf et al., 2013; Matthews et al., 2019; Nijhuis et al., 2022). The relevance of individual differences underscores the necessity of investigating the groove experience across diverse listener profiles, and it calls for ecologically valid audio stimulus sets that appeal to people with diverse music preferences. In order to allow for generalizations and comparisons across studies, these stylistically diverse stimulus sets should be created with unified and high standards of audio production.

Many investigations focused on which and how music-related measures impact groove. It is hardly possible to discuss all of them in a single study, but we can group them according to the three different types of data they are based on: symbolic representation such as music notation (e.g., for syncopation), MIDI data (e.g., for microtiming or tempo), and audio (e.g., for pulse clarity or timbre-related aspects). Accordingly, a stimulus set is most useful if it includes data that allows for the calculation and, in best case, manipulation of measures in all three formats. Additionally, the psychological model of groove (Senn et al., 2019, 2023a, b, 2024; Bechtold et al., 2025c; Jerjen et al., preprint), a recent psychological theory of groove, showed that four cognitive processes (pleasure, energetic arousal, time-related interest, representation of temporal regularity) influence the experience of groove. To facilitate the investigation of groove model-related hypotheses, the stimuli set should also include these behavioural measures alongside data on urge to move.

Existing music research corpora differ in content and format, and they determine the types of research questions that can be studied. The Essen Folksong Collection (Schaffrath, 1995) contains 6,213 monophonic folk tunes; and the Bach Chorales (Hentschel et al., 2023) presents 371 four-part chorales by J. S. Bach. Both include only symbolic data and are used in computational musicology. The Billboard Corpus (Burgoyne, 2012; Harrison & Pearce, 2018) covers 890 pop songs with form and chord annotations, but includes neither audio (due to copyright) nor musical notation. In contrast, the MediaEval Database for Emotional Analysis in Music (DEAM, Aljanaki et al., 2017) has 1,802 royalty-free 45-s excerpts with audio, time-continuous arousal and valence ratings, and feature analyses, making it suitable for perceptual and affective research.

Re-using stimulus corpora across studies allows for the comparison and replication of results, and cumulative progress. Currently, groove research in particular and music psychology more broadly often rely on ad hoc stimuli sets. This makes it difficult to reproduce measurements or compare results across studies—even though some sets, such as those from Witek et al., (2014), Matthews et al. (2019), and Senn et al. (2020), were used in several studies. A large, diverse corpus of stimuli would be a valuable resource for groove research, enabling systematic analysis of rhythmic and movement-inducing properties across many musical examples. Just as neuroimaging relies on standardized face databases (Gronenschild et al., 2009) and visual perception research on well-normed object image sets (Snodgrass & Vanderwart, 1980) to ensure comparability across laboratories and decades, a well-documented, openly accessible stimulus corpus can serve as a shared standard in groove research (or music psychology), fostering reproducibility, comparability, and cumulative knowledge.

In summary, the characteristics and requirements of a widely usable corpus of stimuli for groove research can be summarized as follows:

- A maximum number of stimulus properties can be controlled and manipulated by the researchers.
- The music is ecologically valid, i.e., it sounds like real music to listeners but does not introduce copyright issues for the study.
- The corpus is sufficiently large to allow for the selection of stimuli using different criteria, such as music from different genres, multiple stimuli from the same genre, music from different decades, different tempi, etc.
- The accompanying data provides behavioural ground-truth measurements that are commonly used in groove research. This data is available on stimulus level (i.e., aggregated per stimulus) and on observation level (i.e., individual ratings by participants).
- The data further contains some music- and listener-related data, as well as contextual data.

The goal of this study is to provide such a widely usable audio stimulus set with relevant measures. The set (the Lucerne Groove Library) consists of 444 audio-reconstructed bass lines and drum patterns, each of eight bars duration, taken from popular music excerpts. We opted for drums and bass reconstructions for a variety of reasons:

- Bass and drums have been identified as instruments that are relevant to groove as a musical structure (Moore, 2012; Spicer, 2001), and they are fixtures in the instrumentation of all Western popular music styles.
- Both bass and drums have been used in studies about the groove experience. Bechtold et al. (2024), Kilchenmann and Senn (2015), and Senn et al. (2016) used stimuli with both bass and drum patterns. Kawase and Eguchi (2010), Witek et al. (2014), Etani et al. (2018), Senn et al., (2018, 2024), Eaves et al. (2019), Spiech et al. (2022), Seeberg et al. (2024), and Bechtold et al., 2025a) used only drums. Both bass and drums provide low frequencies that have been found important for the groove experience (Cameron et al., 2022b; Hove et al., 2020; Lustig & Tan, 2019; Stupacher et al., 2016; Wesolowski & Hofmann, 2016).
- The combination of bass lines and drum patterns creates ecologically valid musical textures that may be heard on their own during at least a portion of a song.
- From an earlier project (see Senn et al., 2018), our team already has experience reconstructing drum patterns. These previously reconstructed patterns will also be re-used for the creation of the new stimulus set.
- The electric bass is less complicated to reconstruct than other instruments, e.g., guitars or vocals.

## Methods

### Stimuli creation

#### Previous corpus and expansion

We published the first version of the Lucerne Groove Library in tandem with an earlier study (Senn et al., 2018). The first version encompassed 248 drum patterns eight bars in length reconstructed from commercially available Western popular music recordings. The selection process for the patterns relied on a simple method consisting of first selecting 50 famous drummers and then five drum patterns played by each of these drummers, resulting in a selection of many iconic drum pattern stimuli. The current study’s expansion of the previous corpus consisted in two steps: firstly, we added bass lines to the drum patterns of the existing corpus. Secondly, we selected additional patterns based on the renown of the bassist and the famousness of the bass line and reconstructed both the drum pattern and the bass line. This resulted in a total of 444 combinations of drum patterns and bass lines.

#### Selection

In order to select the patterns based on the bass line, we first compiled a list of famous bass players. We collected names by asking professional bass players and bass educators, consulted online resources, magazines, and research articles. This resulted in a list of 246 bass players that we ordered by number of mentions in the consulted sources (between 1 and 22). We excluded bass players that mainly played jazz and double bass (e.g., Charles Mingus and Ray Brown), because their improvised walking lines follow different musical principles from the composed or at least semi-repetitive bass patterns that we focused on. Including double bass would also introduce a separate instrument with distinct timbral and articulatory properties, which could confound comparisons. We then selected the top 50 of the remaining bassists on the list, and five songs for each of these bass players. In several cases, the bassist played in a band with a drummer from the initial corpus, e.g., drummer John Bonham and bassist John Paul Jones of Led Zeppelin, or drummer David Garibaldi and bassist Rocco Prestia of Tower of Power. For these bass players, we judged how many of the bass lines in the original corpus were representative of their work and reduced the number of newly selected stimuli accordingly. However, we always chose at least two new stimuli specifically for the bass line. With these overlaps reducing the new material, we selected eight-bar passages from 198 songs for the expansion of the corpus that featured a famous, iconic, or simply style-typical and well-played bass line while avoiding bass solos. The consulted sources for player selection, online forums for bass players, and chart placement provided valuable help for selecting the songs, but sometimes the familiarity of the authors with a bass player’s repertoire led to a pragmatic choice. The selected passages had to be in 4/4 (or 12/8) meter, avoid playing techniques we cannot reconstruct, and be acoustically clear. Repetitive patterns (including variations) were preferred, though some less repetitive sections were also chosen. We selected the eight-bar segments from different formal sections—introductions, choruses, verses, interludes, (non-bass) solos—without systematically varying this parameter. Since these rules sometimes allowed for flexibility, the selection of songs and sections was influenced by the authors’ subjective views of what counts as well-played music or representative of a style, band, or individual player. In two cases, this led to two different sections of the same recording in the corpus, e.g., the Beatles’ “Come Together” for which we had the solo section in the previous corpus but wanted to include the iconic bass line in the corpus expansion. For each excerpt, we notated the song, artist, recording year, and location, and we time-stamped the start and end time of the selected passage in the version of the original recording we used (mostly obtained on iTunes). For each bass player and drummer, we also retained their full name, alias if applicable, birth year, and the country where they grew up.

#### Transcription and timing measurements

After the selection of the excerpts from the original recordings, we collected the necessary data for the reconstruction. As in the initial version of the corpus, we did this in two steps: (1) manual transcription of the music to identify and categorize all played events and (2) measuring the exact timing of each event. A professional drummer (author 3) transcribed the drums, and a professional bass player (author 2) transcribed the bass lines. Both transcriptions were checked afterwards by author 1, also a professional musician. The transcriptions included playing techniques (e.g., slap, plectrum, or harmonics for bass; rim clicks, fingers, or brushes for drums) and dynamics in four levels (1 = accent, 2 = loud, 3 = soft, 4 = ghost note). Some aspects were simplified in the transcriptions; for example, we did not measure and reproduce the exact pitch of the toms for each of the recordings. This kind of reduction limited the number of required drum instruments to 24. Some additional percussion instruments were included if they were deemed important for the drum pattern, even though they might have been performed by an additional percussionist (e.g., the shaker in Michael Jackson’s “Billie Jean”). After creating and checking the transcriptions, we measured the timing of each onset manually one by one using the Lucerne Audio Recording Analyzer software (LARA, version 2.6.3). Based on the transcriptions, all events were first marked roughly in a spectrogram and subsequently measured exactly in an oscillogram. Drums, due to their loud and percussive nature, are relatively easy to measure exactly. Cymbal onsets with their strong high-frequency content could easily be located using a high-pass filter on the audio. We are confident that the respective measurements are within a 5-ms error margin. For the bass, timing measurements proved to be more difficult: electric bass notes have a relatively long onset transient that makes it difficult to locate the exact beginning of a note, especially if other instruments play at the same time. For this reason, we (partly) separated the instrument stems with Izotope RX 8 (version 8.1.0.544) in some cases and took the measurements from an audio file that only included the bass (aside from some artefacts and other bass instruments). With this approach, we are confident that our bass onset timing measurements are within a 15-ms error margin. In total, we measured 85,941 events across the 444 clips (59,903 events for the drums, 26,038 the bass). For each of them, we stored the timing data (in seconds), the rhythmic position within the eight bars (in quarter notes), name of the instrument, dynamics level, and playing technique. For bass, we also stored pitch and rhythmic event duration. These two values were not measured (i.e., no measurement of note offset or average event pitch) but based on the transcriptions (durations were considered carefully during the transcription process).

#### Reconstruction

Based on the measured data, we created a MIDI file for each stimulus that contained all events and respective information in coded form (e.g., legato articulation and other playing techniques are triggered by silent MIDI events on specific pitches). As the bass samples had a long rise time, we applied a timing offset of −30 ms on all bass measurements for the reconstruction (−20 ms for slap playing style). The resulting MIDI files were imported into the Reaper digital audio workstation (version 6.14) for the reconstruction based on audio samples. In contrast to the first version of the corpus which features “neutral” audio drum samples, we decided to create five different, style-typical audio sample sets per instrument, i.e., five sets for bass and five for drums (Table 1). This allowed for the reconstruction of most of the patterns with sounds that are at least somewhat close to the original, thus improving ecological validity. The drum audio sample sets were created in Toontrack EZ Drummer 3 (version 3.0.6) and some library extensions, whereas the bass sample sets were created with plugins by Ample Sound (all version 3.1.0). Some audio sample libraries were used for multiple sets, but we used different instruments from these (e.g., different snare drums for pop, rock, and metal drums) or settings (e.g., different equalizers for funk and slap bass) for all sample sets. In addition to the five planned audio sample sets, we created one audio set for brushes and percussion.

**Table 1:** Overview on the audio sample sets that we created for the corpus

Instrument	Set name	Library	Stimuli
Drums	Modern funk drums	Main room	102
	Pop drums	Bright room	99
	Funk drums	Funkmaster Clyde extension	93
	Rock drums	Bright room	86
	Metal drums	Bright room	57
	Brushes	Jazz extension	6
	Percussion	Latin Cuban percussion extension	50
Bass	Funk bass	Ample bass jazz	197
	Slap bass	Ample bass jazz	23
	Metal bass	Ample metal ray	48
	Pic bass	Ample bass precision pick extension	61
	Pop bass	Ample bass precision	109

Stimuli refers to the number of stimuli for which this set was used, with few stimuli having only one assigned sample set because they are drums or bass only. All drum libraries are by Toontrack, and all bass libraries by Ample Sound. The exact pre-sets can be found in the online repository.

For each drum sample set, we individually assigned the intended sounds to specific MIDI pitches. The MIDI mapping for each set of audio samples allowed us to deal with different perceived loudness of individual instruments or audio samples. The pre-sets and MIDI mapping for the audio sample sets can be found in the online repository. With their expertise for the respective instruments, authors 2 and 3 vouched for the quality and ecological validity of the resulting sound. In the next step, these authors assigned an audio sample set to each stimulus according to the similarity between the original sound and the sound of the audio sample set. In consequence, the audio sample sets have been used a different number of times across the entire stimulus set (Table 1).

#### Realization

The tracks featured two or three instruments: drums, bass, and, in 50 cases, additional percussion instruments. We decided not to apply panning to the different instruments in the mix (e.g., we did not assign them a specific location in the stereo space), as some future use cases might be affected by this. However, we varied the loudness of the tracks for each stimulus in order to better recreate the aesthetics of the original. We defined three different mix levels: standard (0.00 dB), low (− 2.00 dB), and very low (− 5.00 dB). With these levels, we could recreate the loudness discrepancies of the original instruments in the reconstructions; for example, when drums were much louder in the original, we put the bass track at − 5.00 dB. Afterwards, we applied the reverb track. As the drum and percussion samples had some built-in room sound and the bass samples were dry, the bass track had a higher send level on the reverb track (+ 3 dB) than drums (+ 1 dB). We used the Dragonfly Hall plugin (version 3.2.10) to create the reverb of a small-sized room with bright acoustics. The exact settings for the reverb can be found in the online repository. When this was completed, we rendered the tracks and saved the music to 24-bit WAV files. Lastly, we normalized the loudness of these files to − 20 LUFS.

#### Quality control

Authors 1–3 listened to all reconstructed stimuli to assess their quality. With 85,941 events measured by hand, errors were to be expected, and often these errors were clearly audible in form of rhythmic or dynamic inconsistencies. Each involved author either gave their approval of the reconstruction as an audibly truthful representation of the original, or flagged inconsistencies as precisely as possible. The respective section, timing, dynamics, or instrumentation was then inspected again in LARA, and measurements or associated data were corrected if found to be wrong. We proceeded with the newly generated MIDI file and reconstructed and realized the stimulus anew for another quality control by multiple authors until all authors approved the reconstruction. If the audio samples or the mix compared to the original recording was flagged as inconsistent, the respective settings were changed, and the result checked again by all authors to find the optimal sound and mix. Especially with respect to the timing measurements, it took several rounds for some stimuli to reach the final form, either because the audio was difficult to measure, or the music on the original recording contained rhythmic inconsistencies (which was verified visually by overlaying wave forms of original and reconstruction). We also dropped two stimuli that contained two drum sets playing different patterns simultaneously, because these conflicted with the technical limitations of our reconstruction method.

### Stimuli data

We collected some contextual data on each of the stimuli: name of the song, artist, drummer and bass player, album, recording year, recording location. We also noted the start (0.5 s prior to the first event) and end (1.5 s after the last event) of the segment in the original recording. Descriptive data of the musical content was calculated and measured based on MIDI or audio.

#### MIDI data

For each of the 444 audio stimuli, we derived 13 structural measures directly from the MIDI files, based on our timing measurements of the drum and bass events (Table 2).

**Table 2:** Brief descriptions of the MIDI data-derived structural measures

Variable	Description
Tempo in beats per minute (BPM)	Based on the linear term of a quadratic regression model predicting the onset timing of each event from its metric position in the eight-bar structure
Tempo stability in BPM^2^	The absolute quadratic term of that regression
Stimulus duration in seconds	Time from the first event’s onset to the last event’s onset in seconds
Number of events	Number of onsets
Event density per second	Number of events divided by stimulus duration in seconds
Event density per beat	Number of events divided by number of beats (32 beats, i.e., eight bars of four quarter beats each)
Unique events	Number of metric positions in which an onset occurs
Unique events per second	Number of unique events divided by stimulus duration in seconds
Unique events per beat	Number of metric positions with events divided by number of beats (32)
Eighth note swing ratio	The averaged inter-onset interval ratio between downbeat and off-beat eighth notes. If the number of eighth notes is less than eight, this measure is NA
Sixteenth note swing ratio	The averaged inter-onset interval (IOI) ratio between first and third sixteenth notes per beat, and second and fourth sixteenth notes per beat. If the number of sixteenth notes is less than eight, this measure is NA
Residual microtiming in seconds	The root mean square error between expected position of each metric position (according to the quadratic regression model predicting the onset timing of each event) and the actually observed placement
Mean subdivision in beats	The most common subdivision in the pattern, i.e., whether the pattern is based on eighth notes, sixteenth notes, or triplets, calculated based on mean IOI between events and tempo

#### Audio measurements

We measured 10 objective audio features of the 444 stimuli in the corpus with the MIR Toolbox (version 1.8.1, Lartillot et al., 2008) in MATLAB (version R2023b). For this analysis, we cut each audio file directly after the last onset for these measurements, because the presence or absence of long decays (e.g., in cymbals) affect the measurements. We selected measures that had been related to the urge to move or groove in previous studies (see Table 3). The exact commands for each measurement can be found in the online repository.

**Table 3:** Measured audio features and previous research that related them to the urge to move or groove

Variable	Description
Event density (MIR)^1,2^	Number of detected events (i.e., peaks) per second
Novelty^2^	Probability of consecutive moments based on different musical properties
Percussiveness^1,3,4^	Average attack slope of all events
Pulse clarity entropy^2,5,6^	Entropy of the pulse clarity (i.e., beat salience). Lower entropy means a clearer pulse
RMS loudness^1,2^	Global energy of the signal
Roll off^2,4^	The frequency (in Hz) below which 85% of the energy are contained, associated with brightness
Sub-band flux no. 2^1,2,4^	Spectral flux for the 50–100 Hz sub-band, associated with fullness
Sub-band flux no. 3^1,2,4^	Spectral flux for the 100–200 Hz sub-band, associated with fullness
Sub-band flux no. 7^1,2,4^	Spectral flux for the 1,600–3,200 Hz sub-band, associated with activity
Sub-band flux no. 9^1,2^	Spectral flux for the 6,400–12,800 Hz sub-band, associated with activity
Zero-crossing rate^,2,4^	How often the signal crosses zero per second, a measure of noisiness

Citation key: ^1^ Stupacher et al., 2016; ^2^ Bechtold et al., 2025b; ^3^ Düvel et al., 2022; ^4^ Bechtold et al., 2025c; ^5^ Spiech et al., 2022; ^6^ Witek et al., 2025

### Listening experiment

We conducted an online listening experiment to gather behavioural response data for each stimulus of the corpus.

#### Participants

We recruited 917 participants (534 males, 379 females, 3 other) on the Prolific platform. They were 34 years old on average (*sd* = 11.598) and resided in South Africa (230), the UK (203), United States (109), Poland (69), Canada (30), Portugal (26), Spain (26), Germany (25), Greece (19), Hungary (17), India (16), Italy (16), Kenya (15), France (14), Netherlands (11), Australia (10), and 26 other countries (> 10). The majority self-identified as music listeners (742), a smaller proportion of the sample indicated being amateur (112) or professional (28) musicians, or music students (28). A small minority (7) were not interested in music. The sample of participants had a low Music Sophistication Index (MSI, Müllensiefen et al., 2014) Training score (mean = 18.050, *sd* = 10.164) compared to a UK standard population (Müllensiefen et al., 2014). Participants had a mean Goldsmith Dance Sophistication Index (DSI, Rose et al., 2022) Urge to Dance score of 24.134 (*sd* = 6.026), and generally liked popular music genres, with a mean rating of 4.040 (*sd* = 0.849) on a [0,6] Likert-type rating scale. The majority indicated having good English language skills (native speaker = 478, Common European Framework of Reference for Languages [CEFR] levels C = 344, B = 70, A = 25).

#### Measures

We measured six scales that are related to the psychological model of musical groove (Senn et al., 2019, 2023a) using self-report questionnaires. We measured participants’ experienced urge to move (MOV, 3 items) and their listening pleasure (PLE, 4 items) with the Experience of Groove Questionnaire (EGQ, Senn et al., 2020, 2024). We measured participant’s energetic arousal (ENE, 4 items), their rhythmic interest (INT, 3 items), and their inner representation of temporal regularity (REG, 4 items) with the questionnaires from Senn et al. (2023b). All items were answered on 7-point Likert agreement scales with range [0,6], and the respective item ratings were averaged to form the variables MOV, PLE, ENE, INT, and REG that can be analysed individually, in parts, or together in the SEM version of the groove model (Senn et al., 2023a). Participants also indicated their familiarity (FAM) with the stimuli by answering whether they had heard the music before (one item 5-point Likert scale: definitely not, probably not, I do not know, probably yes, definitely yes).

As in previous groove studies carried out by the authors, participants filled out the MSI Training scale (Müllensiefen et al., 2014) and the DSI Urge to Dance Scale (Rose et al., 2022). Participants also indicated their liking for 21 music styles and assigned each stimulus to one or more musical styles (optional task). We used a weighted calculation to assign each stimulus a style that is based on observed and expected style assignments (for details, see the supplementary of Senn et al., 2021).

#### Procedure

We followed the procedures outlined in Senn et al. (2024) and Bechtold et al. (2025c), but we adapted the randomization scheme such that all stimuli were rated by approximately the same number of participants and always appeared in different combinations. We set up the questionnaire on the SosciSurvey platform (www.soscisurvey.de). The participants were informed about the study and gave informed consent by mouse click. Participants answered the demographic questions and indicated their music preferences. Then they heard an example to adjust the playback volume to a comfortable level. Subsequently, a randomizer selected 10 stimuli, and the six main blocks of the experiment began. In the first block, the participants heard the 10 stimuli (one per survey page), indicated how familiar they were with the music (FAM), and optionally assigned the music to a style. In the second block, participants heard the same stimuli again, but rated the urge to move (MOV) they experienced in response to the music (3 items). In blocks three to six, participants heard the stimuli again and filled out the questionnaires for pleasure (PLE), time-related interest (INT), energetic arousal (ENE), and inner representation of temporal regularity (REG). Which scale appeared in which block was randomised. Between blocks, participants filled the Goldsmith Music Sophistication Index Training Scale and the Goldsmith Dance Sophistication Urge to Dance Scale. After the sixth block, participants received some information about the study. On average, participants needed 36 min to complete the study. They were remunerated with GBP 6.

#### Statistical analysis

We primarily conducted descriptive analysis across several measures to characterise the corpus. We used R (version 4.4.2) in the RStudio environment (version 2024.09.1) and created all figures with the ggplot2 package (Wickham, 2016; version 3.5.1). A more thorough analysis of the data within the groove model framework (Senn et al., 2023a) will be published later in a separate article.

## Results

As the goal of this article is to describe the corpus and its characteristics, this section presents descriptive statistics on stimulus level. However, data on the level of the single observations is available in the online repository for further use.

### Behavioural data

**Fig. 1:**
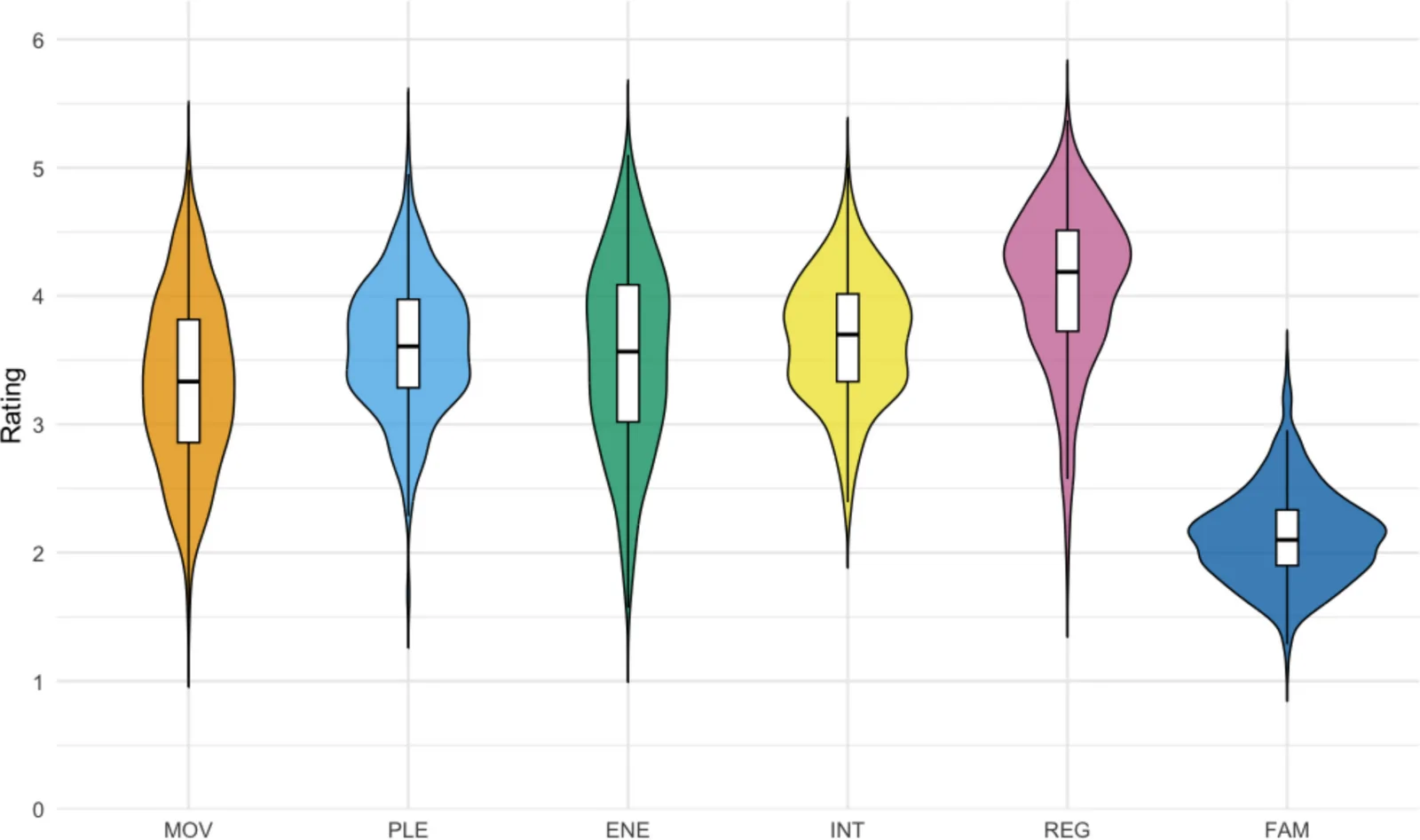
Violin and box plots of the six behavioural measurements in the listening experiment. FAM was rated on a scale from 0 to 4, while all others were rated on a scale from 0 to 6

We collected behavioural data in response to the stimuli in six scales. As shown in Fig. 1, there is mostly a well-centred spread (coefficients of variations are between 0.133 and 0.201) on all scales on stimulus level, but low values are rare. The scales’ skewness and kurtosis (Table 4) indicate that the distributions are acceptable for modelling (Hair et al., 2022).

**Table 4:** Overview of the behavioural data collected in the listening experiment

Variable	Mean	*sd*	Max	Min	Skewness	Kurtosis	*r*_MOV_	*p*_MOV_
Urge to move	3.330	0.668	4.983	1.483	− 0.014	− 0.542		
Pleasure	3.614	0.518	5.206	1.667	− 0.184	0.414	0.743	<.001
Energetic arousal	3.531	0.729	5.100	1.571	− 0.236	− 0.511	0.823	<.001
Time-related interest	3.672	0.488	5.000	2.270	− 0.178	− 0.182	0.698	<.001
Temporal regularity	4.080	0.608	5.369	1.810	− 0.806	0.711	0.286	<.001
Familiarity	2.129	0.363	3.477	1.100	0.468	0.556	0.450	<.001

The scale for the variables is 0–6, except for Familiarity (0–4). Kurtosis is excess kurtosis. The last two columns give the Pearson correlation coefficient and respective *p* value for the variable’s correlation with urge to move (MOV) on stimulus level.

All behavioural ratings are positively correlated with the urge to move (*p* <.001 each). Energetic arousal, pleasure, and time-related interest are strongly correlated with urge to move, while familiarity and temporal regularity show weak to medium strength correlations on stimulus level.

Data for each scale was collected in a separate block. In earlier studies (Senn et al., 2020; Düvel et al., 2021; Kawase et al., 2025; Senn et al., 2023a; Jerjen et al., preprint), several scales were collected in the same block, which led to high positive correlations between the scales. In this study, correlations with MOV on observations level are considerably lower: *r*_PLE_ = 0.519, *r*_ENE_ = 0.549, *r*_INT_ = 0.468, *r*_REG_ = 0.178, and *r*_FAM_ = 0.251 (*p* <.001 each). The reduced strength of these correlations is in accordance with studies that collected the data for each scale in separate blocks (Bechtold et al., 2025c; Senn et al., 2024).

### Stimuli data

#### Styles

Participants optionally assigned each stimulus to one of 21 styles. Based on these answers, every stimulus was assigned to one style (Table 5); 14 styles were assigned at least one stimulus. Rock, funk, pop, jazz, and rock ‘n’ roll form the largest groups, but all styles except rap/hip-hop encompass sufficient stimuli with a variety of urge to move ratings for future selections based on style.

**Table 5:** Frequency of stimuli per style and their respective urge to move ratings

Style	Stimuli	Mean _MOV_	*sd*_MOV_	Max _MOV_	Min _MOV_
Alternative/indie	28	3.222	0.743	4.540	1.889
Blues	32	2.868	0.593	4.333	2.050
Country/western	22	3.326	0.763	4.603	2.150
Dance/electronic	21	3.494	0.623	4.550	2.233
Disco	25	3.800	0.657	4.717	2.067
Funk	57	3.421	0.642	4.900	1.800
Heavy metal	16	3.116	0.473	3.767	2.367
Jazz	41	3.127	0.505	4.067	1.841
Pop	41	3.631	0.668	4.983	2.032
Rap/hip-hop	9	3.065	0.427	3.714	2.467
Reggae/dub/dancehall	20	2.787	0.597	3.817	1.483
RnB	20	3.014	0.625	4.016	2.100
Rock	71	3.418	0.598	4.591	1.587
Rock ‘n’ roll	41	3.585	0.605	4.750	2.333

#### MIDI data

We calculated 13 variables based on the MIDI data of the stimuli. Table 6 gives descriptive data of these measures and their correlation with the urge to move scale.

**Table 6:** Descriptive statistics of the 13 MIDI data-derived variables and their correlation with the urge to move scale on stimulus level

Variable	Mean	*sd*	Max	Min	*r*_MOV_	*p*_MOV_
Tempo in BPM	117.097	31.934	314.650	55.000	0.481	<.001
Tempo stability in BPM^2^	− 0.448	1.709	6.832	− 10.141	− 0.007	.883
Stimulus duration in seconds	17.469	4.409	36.126	5.983	− 0.553	<.001
Number of events	193.054	52.891	398	49	− 0.132	.005
Event density per second	11.419	3.104	23.102	2.979	0.344	<.001
Event density per beat	6.033	1.653	12.437	1.531	− 0.132	.005
Unique events	93.241	27.073	235	37	− 0.243	<.001
Unique density per second	5.452	1.376	10.059	2.381	0.223	<.001
Unique density per beat	2.914	0.846	7.344	1.156	− 0.243	<.011
Eighth note swing ratio	1.093	0.306	3.096	0.853	− 0.012	.798
Sixteenth note swing ratio	1.205	0.326	2.800	0.808	− 0.176	.002
Microtiming in seconds	0.015	0.005	0.044	0.001	− 0.354	<.001
Mean subdivision in beats	0.385	0.123	0.500	0.166	0.184	<.001

For eighth note swing, 404 stimuli featured no swing, and only 48 had swing eighth notes (ratio > 1.5). For two stimuli, eighth note swing is not given (NA), because they featured less than eight eighth notes. Forty-two stimuli featured sixteenth note swing (ratio > 1.5), 264 did not; for 138 stimuli, no sixteenth note swing is given (NA), because they featured less than eight sixteenth notes. Microtiming can be grouped for example as follows: we consider 55 stimuli to have loose timing (> 0.02 s), 347 have average microtiming (between 0.01 s and 0.02 s), and 42 stimuli are tight (< 0.01 s). For the mean subdivision, a total of 234 stimuli are based on eighth notes and 184 feature mostly sixteenth notes; 22 are based on eighth note triplets, and four on sixteenth note triplets. The distributions of the MIDI data-derived variables, either numerically as in Table 6 or in groups as above, allows researchers to select stimuli for experiments based on a variety of measures. The corpus includes extremes as well, e.g., very fast and slow tempos, short and long stimuli, as well as rhythmically dense stimuli and stimuli with little rhythmic activity.

#### Audio measurements

We provide 11 MIR measurements based on the audio of the reconstructed stimuli. As Table 7 indicates, most measures show some variety, and several of these are also correlated with the urge to move scale (MOV). RMS loudness has very little variability because we normalized the loudness of the stimuli to facilitate their use in listening experiments.

**Table 7:** Descriptive statistics of the 11 MIR variables and their correlation with the urge to move scale on stimulus level

Variable	Mean	*sd*	Max	Min	*r*_MOV_	*p*_MOV_
Event density (MIR)	2.733	0.806	7.317	0.916	0.171	.003
Novelty	0.049	0.020	0.119	0.010	0.099	.396
Percussiveness	18.717	5.114	39.921	8.544	0.034	1.000
Pulse clarity entropy	0.695	0.069	0.831	0.454	− 0.161	.007
RMS loudness	0.073	0.004	0.084	0.058	− 0.187	<.001
Roll off (Hz)	7,620.576	1,665.494	13,296.210	282.834	0.113	.188
Sub-band flux no. 2	13.725	4.003	27.362	4.641	0.117	.148
Sub-band flux no. 3	18.753	3.956	32.156	10.779	0.193	<.001
Sub-band flux no. 7	3.755	1.285	9.304	0.286	0.218	<.001
Sub-band flux no. 9	3.150	1.218	8.849	0.153	0.197	<.001
Zero-crossing rate	1,101.714	513.186	3,772.307	152.948	0.179	.002

*p*-values are Bonferroni-corrected for multiple parallel tests.

#### Contextual data

**Fig. 2:**
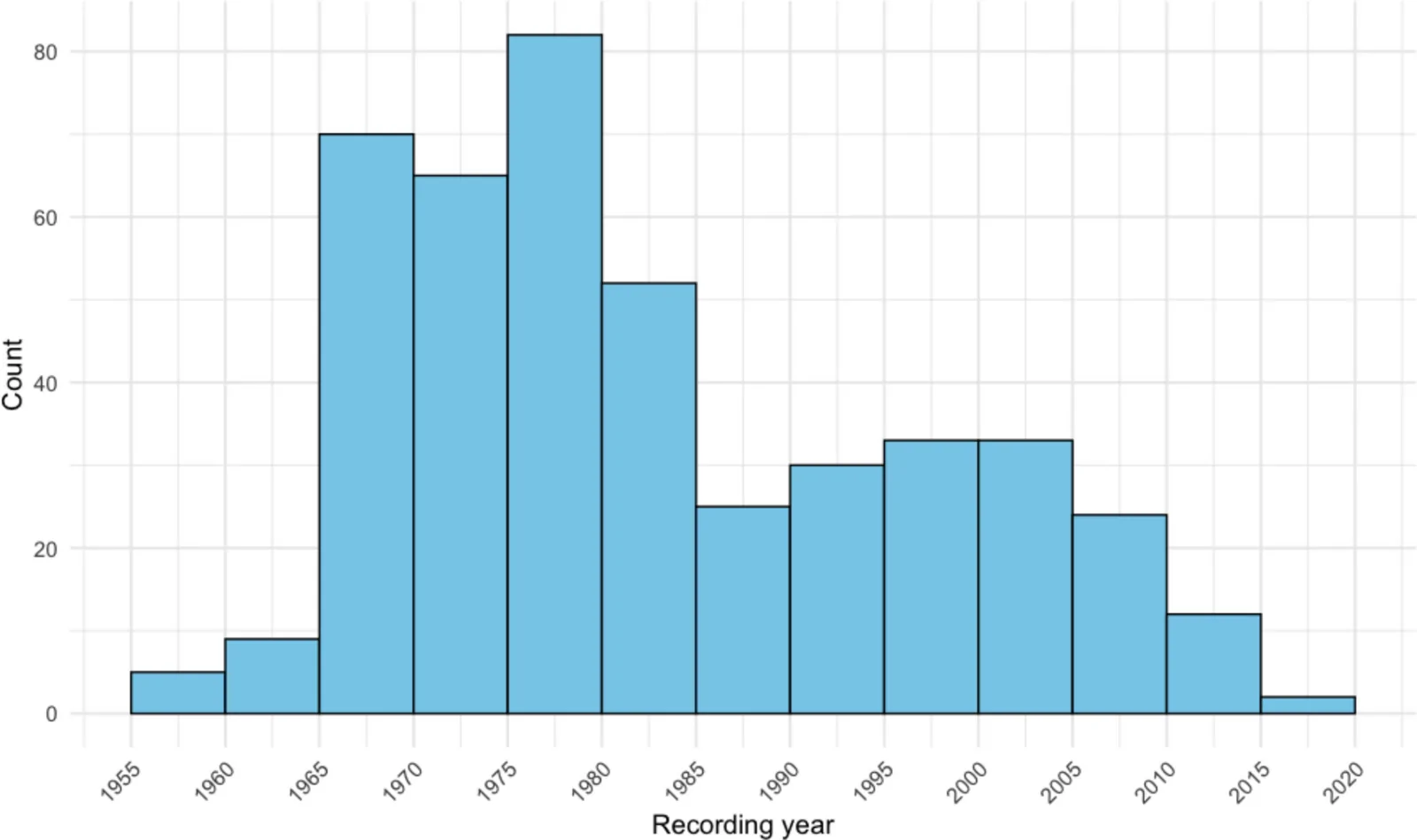
Histogram of the stimuli according to recording year grouped in half-decades

The stimuli cover an extended period of popular music history: 1956–2017 (mean = 1983, *sd* = 13.7 years). Two stimuli have no associated recording year, as the album was recorded over multiple years. Figure 2 shows the distribution of stimuli over half-decades. Stimuli between 1965 and 1985 are strongly represented. For the earliest (1955–1965) and latest (2015–2020) years, we selected only a few songs. The recording year is not correlated with the urge to move (*p* = 0.535) on stimulus level.

The corpus features stimuli as played by 117 different drummers and 119 bass players. The musicians who were featured most often are drummer Ringo Starr (9 stimuli) and bassist Pino Palladino (11). The corpus represents the music of 177 acts/bands, with highest frequencies for James Brown (10) and The Beatles (9). The extracts were from 350 albums, several of which are featured multiple times. Jimi Hendrix’s album “Are You Experienced?” and the Pixies’ “Doolittle” topped this chart with four appearances each.

## Discussion

The method of selecting popular music excerpts and reconstructing the drum patterns and bass lines worked well. With only two exceptions (two simultaneously played drum sets), we were able to successfully measure the timing of all events with reasonable accuracy and realize satisfactory reconstructions of the selected drumbeats and bass lines. The procedure resulted in a corpus of audio stimuli that can be widely used in listening experiments across the human sciences and particularly in groove research. As we provide not only the finalized stimuli, but also MIDI and digital audio workstation files, the stimuli can be manipulated easily by other researchers to suit the needs of their studies. Additionally, the MIDI basis alleviates potential copyright issues related to the original recordings. Our team has already successfully conducted two studies, in which we selected stimuli from the new corpus and manipulated specific variables: in Bechtold et al. (2025c) we tested the effects of different timbres on groove, and in Jerjen et al. (preprint) we studied the effects of tempo variation on groove. Two additional studies by different research teams that also use the new stimulus set are already on the way, which confirms the demand for this kind of corpus.

Besides being controllable and easily manipulable, we aimed for stimuli that sound ecologically valid. Unfortunately, we did not collect data on whether participants found the stimuli to sound naturally. Yet, as three professional musicians (including an expert for each bass and drums) worked in the stimuli creation and realization process, we are confident that the stimuli have a high degree of ecological validity.

The observed behavioural variables, most notably the experienced urge to move, can be used as ground truths for selecting stimuli for future listening experiments that investigate either these variables or how they affect others. The distribution of styles in the corpus makes it suitable for future use cases: 13 styles occur often enough for studies focusing on music from this single style (*N* ≥ 20)—and selection for varying or comparing styles is obviously possible as well. For example, subsets of the corpus could be high and low groove funk, or rock and pop stimuli with similar groove levels. However, one disadvantage is the lack of musical styles that use programmed drums and bass (e.g., EDM): Since we selected stimuli based on human drummers and bass players, programmed popular music styles are not represented. Selecting only music in 4/4 (or 12/8) meter also somewhat limits the musical variety, but most popular music actually uses 4/4. For dedicated studies of odd meters, the corpus would have to be extended in that regard. The decision to create a stimulus set based on Western popular music implies that other musical styles, their respective culture, and musicians are not represented in the stimulus set. This implies that results obtained using this stimulus set should not be generalized beyond the stylistic scope of the stimuli used in this study. Similarly, the behavioural data was largely provided from Western and South African participants. The under-representation of certain cultural groups (e.g., Asia, Middle East and North Africa [MENA]), age groups (60 years and older), or other segments (individuals with high music sophistication) means that ratings and genre classifications should be interpreted with caution when investigating these populations, and we cannot assume that the data are equally valid across all potential samples. They are thought to provide rough orientation to researchers, but they cannot be considered to be an anthropological ground truth.

Stimulus selection for future studies can be based on other variables besides style or behavioural measurements. For example, we provide 13 MIDI data-derived measurements that can be used for stimuli selection: specific tempo ranges, durations, or densities, featuring swing eighth notes or not, being played loose or tight, having an eighth note, sixteenth note, or triplet subdivision as base. Both the MIDI and the audio can be modified, which results in a vast number of ways to manipulate the stimuli in order to vary variables of interest (as exemplified with tempo by Jerjen et al., preprint). We also provide 11 MIR measurements directly from the audio. These can be used as selection criteria, but they also show the considerable breadth of the corpus even though the stimuli only feature drums and bass. Despite this limited instrumentation, we find large differences in brightness, fullness, or activity (Alluri & Toiviainen, 2010) or pulse clarity. Researchers may also choose to make their own measurements using the MIR toolbox or similar plugins and applications.

Of the contextual data, the recording year has probably the greatest interest. The corpus covers seven decades since 1955, but the distribution across decades is very uneven. Music between 1965 and 1985 is strongly represented, and the three decades between 1985 and 2015 are featured reasonably often. Hence, it is a historical corpus of stimuli that starts with the rise of rock’n’ roll, and ends in 2015, with a clear focus on the 1960 s, 1970 s, and 1980s. This is not unexpected: the best-of lists we consulted (created by musicians, magazines, or websites) have a bias towards historical music, i.e., towards legendary bands and musicians that had a strong influence on popular music history. In today’s popular music business, the individual bass and drum players have lost some of their importance, because popular music bands with a fixed line-up of band members are not as important as they used to be.

## Conclusion

We created a corpus of 444 Western popular music drumbeats with simultaneous bass lines of eight-bar length based on patterns recorded by famous drummers and bass players. We publish this corpus of stimuli in different data formats (MIDI, WAV, and as Reaper project files [RPP]) and with a set of accompanying behavioural, structural, and audio measurements as well as contextual data in an online repository. This large corpus allows future studies to create stimulus subsets that suit their respective needs. The stimuli combine easy manipulation with ecological validity while avoiding copyright issues, and offer a variety that allows to select stimuli along many different criteria. We are convinced that the corpus constitutes a useful resource for the investigation of groove but also of other topics in music perception and cognition, and that repeated usage of this corpus can increase comparability and replication of studies. In a next step, we will analyse the data of the listening experiment with the psychological model of groove (Senn et al., 2019, 2023a).

## Data Availability

All data for this study and the corpus are available at https://osf.io/sfwcg/.

## Declarations

### Open practices statement

Materials and data are available at https://osf.io/sfwcg/.

The study was not preregistered.
